# Influence of health literacy on health-related quality of life after total hip arthroplasty

**DOI:** 10.1007/s00402-023-05098-0

**Published:** 2023-10-26

**Authors:** André Strahl, Lara Bücker, Ulrich Bechler, Lara Krüger, Christian Ries, Jan Hubert, Frank Timo Beil, Tim Rolvien

**Affiliations:** 1 Division of Orthopaedics, Department of Trauma and Orthopaedic Surgery, University Medical Center Hamburg-Eppendorf, Martinistr. 52, 20246 Hamburg, Germany; 2 Department of Psychiatry and Psychotherapy, University Medical Center Hamburg-Eppendorf, Martinistr. 52, 20246 Hamburg, Germany

**Keywords:** Total hip arthroplasty, Health literacy, Health-related quality of life, Disease-specific knowledge, Regression model

## Abstract

**Introduction:**

Personal knowledge about the own disease, a key component of health literacy (HL), may have a considerable impact on treatment outcomes. The purpose of this study was to investigate whether the patients’ knowledge about the surgical procedure, risks, and aftercare, as well as the satisfaction with the preoperative level of information, has an influence on the health-related quality of life (HRQoL) after primary total hip arthroplasty (THA).

**Materials and methods:**

A total of 176 patients (68.3 ± 10.3 years, 60.8% female) were evaluated. HRQoL was assessed prior to surgery as well as one and twelve months after THA using the 12-item Short Form Questionnaire. Following standardized surgical informed consent, HL was assessed preoperatively using a self-constructed quiz score, while information satisfaction was measured with a single-item rating scale. Sociodemographic and clinical characteristics, including pain (VAS), functionality (WOMAC), and psychological distress (PHQ-4), were also assessed at baseline. Multiple linear regression analyses were performed to examine whether HL, satisfaction with information, age, social class, WOMAC, VAS, and PHQ-4 predict HRQoL at one and twelve months post-surgery.

**Results:**

The average HL quiz score was 23 ± 5.1 out of a possible 33 points. Social class index significantly influenced HL (*p* < 0.001). A weak correlation between HL and age (*r* = 0.23, *p* = 0.01) and no correlation between HL and psychological distress (*p* = 0.868) were observed. One month after THA, physical HRQoL was significantly predicted by the WOMAC index (*p* = 0.031) and subjective satisfaction with information (*p* = 0.022), but not by HL. After twelve months, only the WOMAC was a significant predictor (*p* < 0.001) of physical HRQoL.

**Conclusion:**

Although subjective satisfaction with the patient’s preoperative level of information had a significant effect on the physical HRQoL at one month after THA, the influence of osteoarthritis severity outweighed this effect after twelve months. HL had no direct influence on HRQoL. These results suggest that patient satisfaction, rather than knowledge, predicts HRQoL.

**Supplementary Information:**

The online version contains supplementary material available at 10.1007/s00402-023-05098-0.

## Introduction

Total hip arthroplasty (THA) is a highly successful surgical procedure to restore hip joint function and alleviate pain in patients with end-stage osteoarthritis (OA) of the hip [1]. The quality and survival of THA has steadily improved over the last decades. There have been improvements in surgical techniques, implant materials, and prosthesis design. These developments have resulted in excellent clinical outcomes and patient satisfaction [2]. Today, new procedures and techniques do not always lead to improved outcomes after surgery. Instead, patient-related factors (e.g., anxiety or depression) may play a crucial role on THA outcomes [3].

The personal knowledge about the own disease and its management can have a considerable influence on the patients’ treatment outcome [4]. The ability to access, understand, and apply health information to be aware of what factors influence health and how to treat a particular condition is called health literacy (HL) [5]. Currently, several definitions of this concept exist simultaneously. Their common denominators are the basic patient attributes knowledge, competence, and motivation [5, 6]. In surgery, low HL is directly related to poor outcomes [7–9]. More specifically, patients with low HL have a reduced understanding of discharge instructions and are less satisfied with treatment outcomes. Recent studies reported that low HL is highly prevalent in patients undergoing surgery [9, 10]. Patients with OA and low HL tend to have a lower preoperative expectation concerning their walking abilities after arthroplasty [7], which may have a direct influence on the outcome [11]. Patients with low HL had worse outcomes and were less likely to be satisfied after total knee arthroplasty [12]. Accordingly, patients with low HL also experienced lower health-related quality of life (HRQoL) [4, 13, 14]. Importantly however, HRQoL is also affected by other factors, e.g., sex [14, 15], psychological distress [16, 17], higher age [18, 19], pain [20], or physical function [14].

The concept of HL is essential as patients are required to understand benefits and risks of the surgical intervention, follow the peri- and postoperative medical instructions and take responsible decisions for their own health [8, 10]. In the broader sense, HL may also involve subjective criteria such as satisfaction with the level of information. Despite its importance, HL is still poorly investigated in patients undergoing orthopaedic surgical procedures (e.g., THA). Hence further investigation is needed [4, 9]. We hypothesized that objective and subjective HL are independent predictors of HRQoL after THA. The study aimed to examine the influence of patients’ knowledge about surgery, risks, and aftercare, as well as their satisfaction with preoperative information, on the HRQoL in patients with OA following THA.

## Methods

### Study design and patients

The study was conducted as single-center study including patients with primary end-stage hip OA scheduled for unilateral THA in our clinic. The data were obtained from the hospital's own knee and hip registry introduced stepwise from 2020. After patient’s consent, clinical data from the medical record supplemented by patient-reported outcome measures (PROM) were collected. The aim of this registry is the documentation of therapeutic interventions, improvement of treatment quality through standardized documentation and evaluation, and the provision of clinical data for research. The registry provides patient data in a pseudonymized form for research purposes in accordance with data protection regulations. The study conformed to the principles of the Declaration of Helsinki [21], and was approved by the local ethics committee (PV7275).

The indication for THA was primary OA as diagnosed by self-reported clinical symptoms, physical examination, and routine radiographs of the hip. Patients with tumors, severe hip dysplasia, trauma, or revision THA were excluded. THA was performed by senior orthopaedic surgeons using the posterior approach at our institution. Cementless press-fit acetabular components were used in all patients (Allofit Alloclassic Cup; Zimmer Biomet, USA). Depending on age, bone quality and anatomical morphology, cemented (M.E. Müller, Zimmer Biomet) or uncemented (Avenir, Fitmore; all Zimmer Biomet) fixation of the femoral components was performed.

Data were collected using a self-constructed questionnaire that included disease-specific HL and as well by standardized patient-reported questionnaires preoperatively at admission (*t*_0_), and postoperatively after one (*t*_1_) and twelve months (*t*_2_). Corresponding to an a priori sample size calculation, 176 OA patients undergoing primary THA with complete pre-operative and follow-up protocol were included. Patients with major complications requiring revision surgery were excluded.

### Outcome measures

The primary outcome was the prediction of HRQoL one month and twelve months after surgery by multiple independent predictors. HRQoL was assessed with the Short-Form 12-Item Health Survey (SF-12) scales mental health and physical health [22]. The SF-12 is a commonly used generic 12-item questionnaire with good psychometric properties for the assessment of HRQoL in OA [23]. Weighted sum scales were calculated (range 0–100), whereby a mean value of 50 represents a healthy reference norm and higher values correspond to higher HRQoL. In all patients, the Harris Hip Score (HHS) was determined to evaluate the outcome of the THA regarding functional outcomes [24]. Moreover, pain, psychological distress, and global physical functioning were assessed as PROM predictors. Pain was determined by a visual analogue scale (VAS), quantifying pain intensity within the last week on a 10 cm line representing the range between “no pain” and “worst pain”. Symptoms of psychological distress comprising anxiety and depression were measured by the validated screening scale Patient Health Questionnaire (PHQ-4) [25, 26]. The PHQ-4 is a generic self-report questionnaire, which uses four items to assess anxiety and depression. The overall physical functioning was assessed by the Western Ontario and McMaster Universities Arthritis Index (WOMAC) global score [27]. The global index score encompasses the pain, stiffness, and physical function. Higher values indicate more severe functional limitations.

Patients’ disease-specific HL was assessed using a self-constructed questionnaire depicting the main content of the statutory standardized pre-surgery patient education and informed consent (Supplementary Fig. 1). Prior to the start of the study, a standardized information scheme was established to provide all patients with the same information. All surgeons performing informed consent in our institution were instructed on the scheme and were advised on the relevant information that should be provided to all patients prior to surgery. Overall, this questionnaire contained 33 items on disease-specific knowledge, which cover the areas complications and specific risks, risk of infection, operation technique, and expected survival of THA. The score used three different response formats: Free-text responses, yes–no decision responses, and multiple-choice responses. Additionally, the subjective satisfaction of patients with their perceived level of information was assessed through an established 7-point NRS ranging from 0 (very dissatisfied) to 6 (very satisfied) [28]. Due to the self-constructed questionnaire, a score evaluation was conducted as part of this study. In this evaluation process, several analyses were performed to assess the performance and validity of the quiz score. A missing item analysis was carried out to identify systematic patterns in unanswered questions within the questionnaire, assessing their potential impact on the overall score. Subsequently ceiling and floor effects were investigated to determine whether the questionnaire had ceiling (i.e., maximum score achieved by many participants) or floor effects (i.e., minimum score achieved by many participants). Additionally, the impact of demographic variables such as social class, sex, and age on the quiz scores were investigated.

### Sample size calculation

The estimation of the sample size was performed using G*Power software (ver. 3.1.9.2; Heinrich-Heine-Universität Düsseldorf, Düsseldorf, Germany). With an alpha risk of 0.05, a statistical power of 0.8, and an effect size of *f*^2^ = 0.09 a total sample size of at least 167 patients was required to calculate a multiple linear regression with seven independent predictors. An effect size of *f*^2^ = 0.09 represents a small to medium effect, which is capable to detect significant small beta-weights in the regression model. At the time of the analysis, *n* = 176 patients were included in the patient registry. Accordingly, the number of cases is adequate to achieve sufficient statistical power.

### Statistical analysis

To identify pre-operative predictors of health-related mental and physical HRQoL after THA, multiple linear regression analyses were carried out. Multiple linear regression analyses were performed to determine whether patients’ knowledge operationalized by the quiz score, patients’ satisfaction with their level of information, age, social class index, WOMAC, VAS pain, and the PHQ-4 predicted the mental and physical HRQoL one months and twelve months post-surgery. The regression used the “Enter” method to examine the significant impact of all variables simultaneously. To control the prerequisites of the regression analysis, Pearson correlation for continuous and Spearman correlation for nonparametric variables were applied. Correlations among the independent predictors should amount less than 0.7.

In an additional analysis, group comparisons with *t* tests for independent variables or Mann–Whitney-*U* tests were performed for all significant predictors with group thresholds of < 25th percentile versus > 75th percentile. Shapiro–Wilk normality test was applied to determine normal distribution. To analyse whether differences are clinically relevant effect sizes according to Cohen (*d* values: 0.2–0.5 small effect, 0.5–0.8 medium effect, > 0.8 large effect) were calculated.

Additionally, the long-term improvement of the PROMs applied was evaluated using one-way repeated measures ANOVAs with the effect size partial eta squared (*η*^2^_partial_≈0.01 small effect, *η*^2^_partial_≈0.06 medium effect, *η*^2^_partial_≈0.14 large effect) to determine significant differences among measuring points. With additional repeated measures analyses of covariances (ANCOVA), the influence of HL and the subjective satisfaction with the level of information on the long-term improvement of the PROMs were investigated. Continuous variables are expressed as mean ± standard deviation (SD), while categorial variables are expressed as number and percentage. SPSS statistical program 29.0 (SPSS, Chicago, IL) and GraphPad Prism 7 (GraphPad Software, La Jolla, CA) were used for statistical analyses.

## Results

### Baseline characteristics

Most patients were female (60.8%; *n* = 107), with a mean age of 68.3 ± 10.3 years. Furthermore, most patients (46.0%) completed 9 years of formal school education, completed an apprenticeship as highest vocational qualification (61.4%) and were currently retired (in receipt of a pension; 71.6%) at the time of the study. Following a sociological class index calculation [29], patients were classified to the lower (27.8%), middle (43.8%) and upper class (28.4%). Further baseline characteristics are presented in Table 1.

**Table 1:** Baseline characteristics and demographic data of the included patients

Baseline characteristic	*N* (%), mean ± SD	Range
Participants	*N* = 176	
Sex		
Male	69 (39.2%)	
Female	107 (60.8%)	
Age (years)	68.3 ± 10.3	41–90
VAS pain	6.9 ± 1.8	0–10
HHS	46.4 ± 14.2	6–81
Charnley classification		
A	75 (42.6%)	
B	51 (29.0%)	
C	50 (28.4%)	
PHQ-4 depression/anxiety		
No symptoms	135 (76.7%)	
Yellow flag (6–8 points)	20 (11.4%)	
Red flag (9–12 points)	21 (11.9%)	
Formal school education (years)		
9 years	81 (46.0%)	
10 years	54 (30.7%)	
12 years and more	40 (22.7%)	
Missing	1 (0.6%)	
Occupational education		
No vocational qualification	19 (10.8%)	
Apprenticeship	108 (61.4%)	
Technical colleges	23 (13.0%)	
University	21 (12.0%)	
Missing	5 (2.8%)	
Occupation		
Full time	32 (18.2%)	
Half time	16 (9.1%)	
Unemployed/pension	126 (71.6%)	
Missing	2 (1.1%)	
Sociological class index		
Lower class	49 (27.8%)	
Middle class	84 (43.8%)	
Upper class	56 (28.4%)	

*HHS* Harris hip score, *PHQ* patient health questionnaire, *VAS* visual analogue scale

### Evaluation of a quiz on disease-specific knowledge

The score represents the patients’ knowledge of their disease, which is considered the key component of HL. The score was calculated by summing all correct answers; missing items are considered as incorrect answers. In this sample the mean score amounts 23.8 ± 5.1 points (range 3–33 points; median = 25, IQR = 8) of 33 possible points. The social class influenced the achieved quiz score points (*p* < 0.001), whereby both, the lower (*p* < 0.001) and the middle class (*p* = 0.01) differed from the upper class both achieved fewer scores. There were no significant differences in terms of sex (*p* = 0.954). Only a weak correlation between the quiz score and age (*r* = 0.23, *p* = 0.01) and no correlation between the quiz score and psychological distress (*r* = − 0.013, *p* = 0.868) were observed.

Descriptive analysis of the score demonstrated a left-sloped, non-normal distribution. The Shapiro–Wilk test confirms the non-normal distribution (Shapiro–Wilk _df 175_ = 0.925, *p* < 0.001). No floor or ceiling effects were observed due to less than 15% of the patients reached the highest (0.6%) or lowest (0%) possible score. Since the score contains different answering formats, no reliability or discriminatory item analysis could be calculated. However, there was sufficient variability in the response scales of less than 80% marks in one response category. On average, we detected 74.1% patient’s responses in one item category over all items.

### Longitudinal changes in outcome measures

**Fig. 1:**
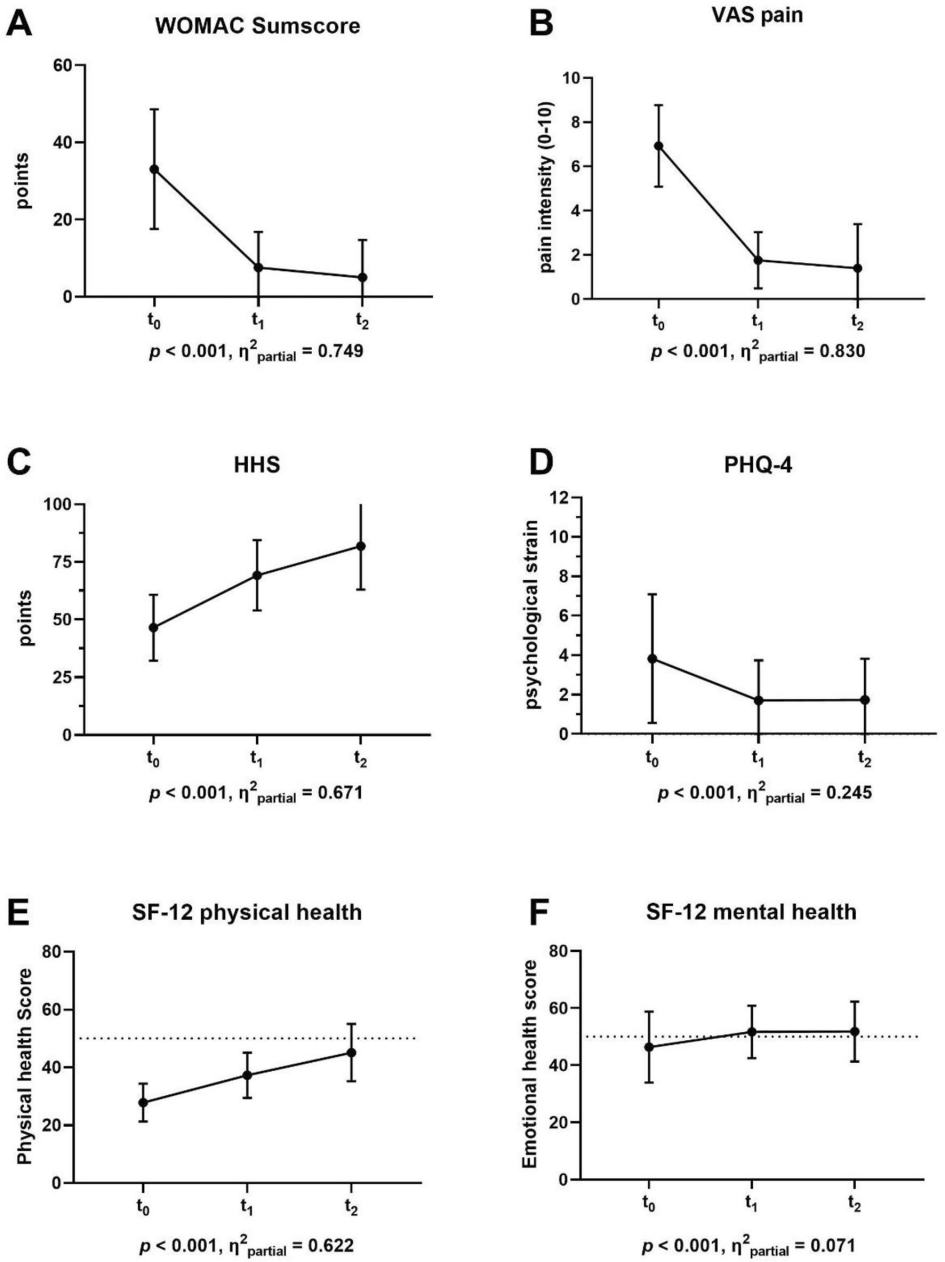
Repeated measures ANOVA analyses for improvement of patient reported outcome parameters from baseline to one and twelve months after THA surgery. At each measurement point, mean ± standard deviation is displayed for the corresponding score. **A** Western Ontario and McMaster Universities Osteoarthritis Index (WOMAC), **B** Visual analogue scale (VAS) for pain, **C** Harris Hip Score (HHS) score, **D** Patient Health Questionnaire (PHQ)-4, **E** 12-Item Short Form Survey (SF-12) for physical health related quality of life, **F** 12-Item Short Form Survey (SF-12) for mental health related quality of life

At twelve months, THA patients showed decreased pain (*p* < 0.001, *η*^2^_partial_ = 0.83) and improved HHS scores (*p* < 0.001, *η*^2^_partial_ = 0.671), indicating the functional efficacy of the surgery. In line with these results, the WOMAC index demonstrated a decrease over time (*p* < 0.001, *η*^2^_partial_ = 0.749). Anxiety and depression symptoms were reduced twelve months after surgery (*p* < 0.001, *η*^2^_partial_ = 0.245). Before surgery, 11.9% of the patients had noticeable high psychological distress, while the proportion reduced to 1.6% 1 year after. Concerning HRQoL, the main effect of time twelve months after THA was significant for the SF-12 primary outcomes physical (*p* < 0.001, *η*^2^_partial_ = 0.622) and mental health (*p* < 0.001, *η*^2^_partial_ = 0.071) (Fig. 1A–F). The larger effect size for physical health indicates that HRQoL is more affected by physical symptoms than psychological distress in the overall collective. The results of the additional repeated measures ANCOVA revealed that neither the quiz score nor the patients’ satisfaction with their level of information had an impact on the longitudinal change of clinical outcome parameters (Table 2).

**Table 2:** Analysis of covariance for indirect effects of the quiz score and patient’s satisfaction with information on the improvement of patient reported outcome parameters from baseline to one and twelve months after THA surgery

Outcome variable	Confounder	Target variable	Mean square	*F*	*p* value	*η*^2^_partial_
Indirect confounding effects of the quiz score						
WOMAC sumscore	Quiz score	WOMAC sumscore change over time	14.801	0.104	0.901	0.002
VAS pain	Quiz score	VAS pain change over time	2.967	1.246	0.294	0.034
HHS	Quiz score	HHS change over time	20.754	0.123	0.884	0.003
PHQ-4	Quiz score	PHQ-4 change over time	2.414	0.788	0.458	0.018
SF-12 physical health	Quiz score	SF-12 physical health change over time	1.481	0.028	0.972	0.001
SF-12 mental health	Quiz score	SF-12 mental health change over time	219.901	2.830	0.064	0.060
Indirect confounding effects of the patient’s subjective satisfaction with information						
WOMAC sumscore	Satisfaction	WOMAC sumscore change over time	14.705	0.103	0.902	0.002
VAS pain	Satisfaction	VAS pain change over time	5.498	2.309	0.107	0.062
HHS	Satisfaction	HHS change over time	49.912	0.255	0.775	0.006
PHQ-4	Satisfaction	PHQ-4 change over time	2.006	0.654	0.522	0.015
SF-12 physical health	Satisfaction	SF-12 physical health change over time	7.021	0.134	0.874	0.003
SF-12 mental health	Satisfaction	SF-12 mental health change over time	149.487	1.924	0.153	0.042

*HHS* Harris hip score, *PHQ-4* patient health questionnaire-4, *SF-12* 12-item short form survey, *VAS* visual analogue scale für pain, *WOMAC* Western Ontario and McMaster Universities osteoarthritis index

### Satisfaction with information but not quiz score as independent predictor of health related quality of life

The mean subjective satisfaction with the own level of knowledge was rated 4.7 ± 1.1 points on the 7-point rating scale. There were no significant differences in terms of sex (*p* = 0.678) or social class (*p* = 0.966). Also, age demonstrated no association with the subjective satisfaction (*r* = 0.087, *p* = 0.256). Notably, the quiz score did not correlate with the subjective satisfaction with information (*r* = − 0.036, *p* = 0.639). The correlation analyses performed prior to the multiple regression models demonstrated no correlation greater than 0.7 between the independent predictors (Supplementary Fig. 2).

Multiple regression analysis was performed to identify potential independent predictors of mental and physical HRQoL at one month and twelve months after surgery. One month after surgery, the physical HRQoL was predicted by the WOMAC baseline score (*β* = − 0.139, *p* = 0.031) and subjective satisfaction with the received information (*β* = 1.474, *p* = 0.022). The model explained 9% (R^2^) of the variance. These results indicate that increased satisfaction and improved result of the WOMAC significantly improve the physical HRQoL. After 1 year, only the WOMAC at baseline represented a significant predictor for the physical HRQoL (*β* = − 0.3, *p* < 0.001). This model explained 13.5% (R^2^) of the variance (Table 3). Separate regression analyses to predict the mental HRQoL indicated that the psychological distress (PHQ-4) but not HL or satisfaction with information at baseline significantly influenced the mental HRQoL both after one month (*β* = − 0.934, *p* = 0.001) and twelve months (*β* = − 1.182, *p* = 0.001; Table 4). These regression models explained 21.7% (one-month R^2^) or 21.5% (twelve months R^2^) of the overall variance.

**Table 3:** Logistic regression for independent predictors of the SF-12 physical health summary scales one and twelve months after surgery

Predictor	SF-12 _physical health_ 1 month post-op				SF-12 _physical health_ 12 months post-op			
	*β*	*SE β*	*t*	*p* value	*β*	*SE β*	*t*	*p* value
Intercept	31.335	7.835	4.0	< 0.001	47.483	10.7	4.438	< 0.001
Quiz score	0.08	0.154	0.518	0.586	− 0.034	0.205	− 0.167	0.867
Satisfaction with information	**1.474**	**0.635**	**2.32**	**0.022**	0.869	0.887	0.979	0.33
Social class index	0.174	0.419	0.416	0.678	0.762	0.519	1.468	0.145
Age	− 0.050	0.072	− 0.694	0.489	− 0.098	0.095	− 1.024	0.308
WOMAC (baseline)	− **0.139**	**0.064**	− **2.187**	**0.031**	− **0.3**	**0.087**	− **3.443**	** < 0.001**
VAS pain (baseline)	0.606	0.435	1.393	0.166	0.752	0.599	1.256	0.212
PHQ-4 (baseline)	− 0.035	0.27	− 0.128	0.898	0.229	0.36	0.636	0.526
	Overall model evaluation *p*_ANOVA_ = 0.045 Adjusted R^2^ = 9.4%				Overall model evaluation *p*_ANOVA_ = 0.027 Adjusted R^2^ = 13.5%			

Bold font indicate statistical significance (*p* < 0.05)

*ANOVA* analysis of variance, *PHQ-4* patient health questionnaire-4, R^2^ coefficient of multiple determination for multiple regression, R^2^ is the percentage of the dependent variable variation that the linear model explains, *SF-12* 12-item short form survey, *VAS* visual analogue scale für pain, *WOMAC* Western Ontario and McMaster Universities osteoarthritis index

**Table 4:** Logistic regression for independent predictors of the SF-12 mental health summary scales one and twelve months after surgery

Predictor	SF-12 _mental health_ 1 month post-op				SF-12 _mental health_ 12 months post-op			
	*β*	*SE β*	*t*	*p* value	*β*	*SE β*	*t*	*p* value
Intercept	60.499	8.292	7.296	< 0.001	46.221	10.517	4.395	< 0.001
Quiz score	0.13	0.163	0.801	0.425	− 0.173	0.202	− 0.855	0.394
Satisfaction with information	0.704	0.672	1.047	0.297	0.407	0.872	0.466	0.642
Social class index	− 0.082	0.444	− 0.185	0.854	0.3	0.511	0.588	0.557
Age	− 0.079	0.076	− 1.035	0.303	0.113	0.094	1.207	0.23
WOMAC (baseline)	− 0.077	0.067	− 1.136	0.258	− 0.125	0.086	− 1.463	0.147
VAS pain (baseline)	− 0.523	0.461	− 1.134	0.259	0.99	0.589	1.682	0.096
PHQ-4 (baseline)	− **0.934**	**0.286**	− **3.264**	**0.001**	− **1.182**	**0.354**	− **3.34**	**0.001**
	Overall model evaluation *p*_ANOVA_ < 0.001 adjusted R^2^ = 21.7%				Overall model evaluation *p*_ANOVA_ < 0.001 adjusted R^2^ = 21.5%			

Bold font indicate statistical significance (*p* < 0.05)

*ANOVA* analysis of variance, *PHQ-4* patient health questionnaire-4, R^2^ coefficient of multiple determination for multiple regression, R^2^ is the percentage of the dependent variable variation that the linear model explains, *SE* standard error, *SF-12* 12-item short form survey, *VAS* visual analogue scale für pain, *WOMAC* Western Ontario and McMaster Universities osteoarthritis index

**Fig. 2:**
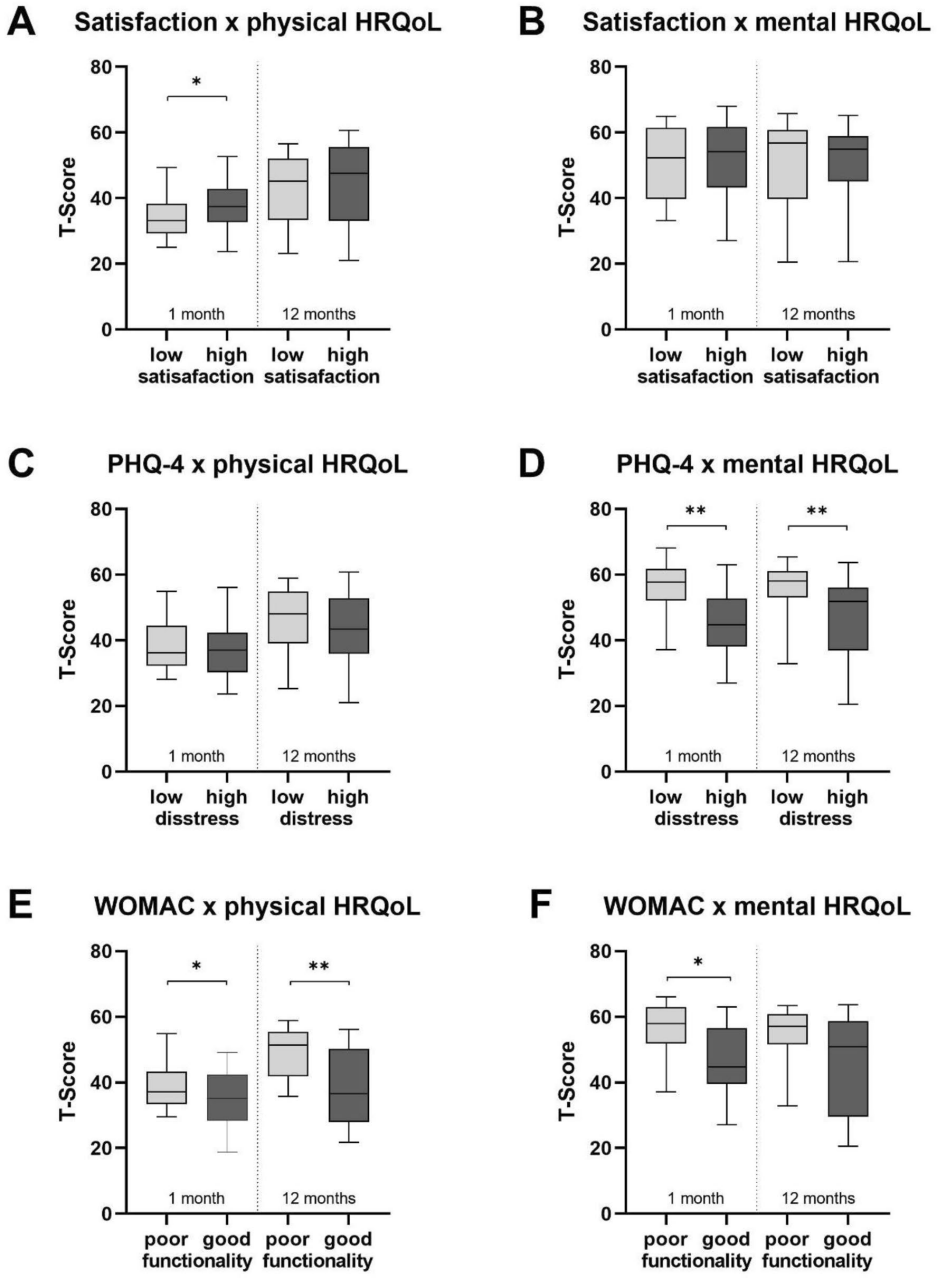
Group comparisons for the impact of high subjective satisfaction, low psychological distress and good physical functionality on the physical and mental health related quality of life (HRQoL). **A** Comparison of patient with low (< 25th percentile) and high (> 75th percentile) subjective satisfaction with their level of information in terms of the post-surgery physical HRQoL and **B** mental HRQoL, **C** Comparison of patient with low (< 25th percentile) and high (> 75th percentile) psychological distress measured with the Patient Health Questionnaire (PHQ)-4 in terms of the post-surgery physical HRQoL and **D** mental HRQoL, **E** Comparison of patient with poor (< 25th percentile) and good (> 75th percentile) functionality measured with the Western Ontario and McMaster Universities Osteoarthritis Index (WOMAC) index scale in terms of the post-surgery physical HRQoL and **F** mental HRQoL

Group comparisons between patients with low and high satisfaction with their received information demonstrated significant differences in physical but not mental HRQoL one month after THA (*p* = 0.025, *d* = 0.592, Fig. 2A, B). As expected from the regression model, patients with lower psychological distress prior to surgery had a significant superior mental but not physical HRQoL both 1 (*p* < 0.001, *d* = 1.264) and twelve months (*p* = 0.003, *d* = 0.986) after THA (Fig. 2C, D). Similarly, patients in good physical condition at baseline showed a better physical HRQoL at 1 (*p* = 0.04, *d* = 0.533) and twelve months (*p* = 0.003, *d* = 1.152) after surgery (Fig. 2E). Moreover, patients with a good WOMAC score also demonstrated a significant better mental HRQoL after one months (*p* < 0.001, *d* = 1.07). This difference could not be replicated after twelve months (Fig. 2F).

## Discussion

In the present study, we investigated the influence of the patients’ knowledge and subjective satisfaction with the preoperative received information (i.e., key parameters of HL) on HRQoL at one and twelve months after THA. HRQoL was chosen as primary outcome because it provides a comprehensive assessment of the patient’s health status that considers both objective clinical measures and subjective patient-reported outcomes. Our holistic approach aimed to address the multidimensional impact of THA on the patient’s well-being. Identifying factors that influence the HRQoL is an important element in the process of treatment optimization, as a high HRQoL is essential for the patients’ long-term functionality, mental health, and an improved overall satisfaction with life. Our study identified subjective satisfaction with the information received and the WOMAC score at baseline as significant predictors of physical HRQoL after THA. Interestingly, the disease-specific knowledge determined by the quiz score did not show a significant influence on the outcome in any of the analyses. Importantly however, preoperative psychological distress (i.e., anxiety and depression) predicted mental HRQoL at one and twelve months after THA. Group comparisons between the 1st and 4th quartile of each significant predictor variable confirmed the results of our regression models. All analysis demonstrated significant differences in terms of HRQoL. At least medium effect sizes according to Cohen were observed. Large effect sizes, and thus also the most importance influence on the HRQoL, were obtained for psychological distress (PHQ-4) and mental HRQoL as well as for the WOMAC score and physical HRQoL.

We observed significant improvements over time in all relevant PROMs, e.g., WOMAC, VAS pain, HHS, PHQ-4, SF-12 physical health and SF-12 mental health. Essentially, our findings indicate that patients experienced positive changes in these important health-related measures within twelve months after THA. These positive effects of THA have long been established and documented in the literature [30–33]. Additional analyses undertaken to investigate the potential influence of HL and subjective satisfaction with information, did not yield statistically significant results concerning their impact on the observed positive changes in PROMs after THA. These findings contrast with the observation that patients with higher HL may be more able to understand and follow postoperative instructions, care for themselves, and manage their recovery effectively [34]. Furthermore, patients possessing higher HL levels may exhibit increased initiative in seeking social support, fostering communication with healthcare practitioners, and participating in activities aimed at enhancing their well-being [35].

### Time-dependent predictors of physical HRQoL after THA

The observed independent predictors of the physical HRQoL were not consistent over time and changed by twelve months after surgery. Although subjective satisfaction had a significant effect on the outcome after one month, the preoperative functional health overpowered this influence twelve months after surgery. Overall, the multiple regression reveals that only 9–13.5% of the variability observed in the target variable was explained by the regression model, indicating that other predictors seem to have a stronger influence on the physical HRQoL. The actual functional status of the patients is apparently more important than the preoperative status. It is interesting to note that the functional status as single predictor has a higher influence on the HRQoL at later measurement time points, not immediately after THA. A previous systematic review has investigated various predictors of HRQoL improvements, with mental health status at baseline, mobility, comorbidities, level of education and the preoperative physical function score having a significant influence on the WOMAC [36]. Accordingly, we assume that immediately after THA, a number of different parameters are accountable for the experienced HRQoL. After consolidation, the functional status emerges as one of the most important predictors of HRQoL.

### Impact of psychological distress on mental HRQoL after THA

In contrast to physical HRQoL, mental HRQoL at one month and twelve months after THA was influenced exclusively by psychological distress at baseline, measured by the PHQ-4. Our results confirm recent studies that have demonstrated that psychological distress influences the clinical outcome after arthroplasty [37–41]. These findings not only suggest that mental health should be routinely assessed prior to arthroplasty but also that preventive measures should be considered in patients with signs of anxiety and depression to improve the outcome of THA.

### Differences between objective knowledge and subjective satisfaction with information as predictors of HRQoL

Overall, our data point to a possible relationship of preoperative satisfaction with the information provided by the surgeon and the short-term perceived physical HRQoL one month after THA, but causality remains to be proven by future studies. The objective disease-specific knowledge does not seem to have any influence on the HRQoL. In this context, it is also noteworthy that no correlation between satisfaction with the level of information and objective knowledge determined by the quiz score could be shown. Nevertheless, HL skills are required to understand surgery procedures, comply with the postoperative instructions and to provide a fully informed consent to surgery [7, 34, 35].

### Strengths and limitations

This study has clear strengths. It is the first study to investigate the impact of patients’ knowledge as core part of HL on the HRQoL in a sample of patients with primary hip OA undergoing THA. Only established and standardized questionnaires were used to examine the clinical outcomes. A limitation can be seen in the self-constructed quiz. In contrast to already established questionnaires on HL [42], we aimed to test HL specific to a cohort of OA patients undergoing THA. A self-developed quiz form on disease knowledge has been previously used in the context of orthopaedic conditions [12, 43]. To the best of the authors’ knowledge, there is currently no existing literature or questionnaire that assesses patient knowledge about THA. Therefore, the disease-specific HL score was developed with the collaboration of clinical practitioners at the hospital for this study. Considering the lack of a standardized tool in the field of arthroplasty, this study adopted the methodology approach employed in a study by Meng et al. [43] and designed a self-constructed quiz score to measure the patients’ knowledge of their disease as part of HL This method was applied to isolate and evaluate the knowledge aspect to gain a more focused understanding of its impact on HRQoL. Within our study, the quiz score demonstrated no floor or ceiling effects, and a large range of 3–33 points to derive the practical validity of the score. A limitation of our quiz score could be the method of summing all correct answers and treating missing information as incorrect, leading to a potential bias. This approach assumes that missing responses are equivalent to incorrect responses. This procedure was only applied to single missing items. If a whole page or the complete questionnaire was not completed, the patient was excluded from the analysis. The bias may arise due to participants who may have omitted specific questions intentionally because they were unsure of the correct answer or lacked sufficient knowledge on that particular topic. Generally, there is a need of an integrated theoretical model and measurement instrument that includes and combines HL, patient expectations, and clinical outcomes. A further limitation of our study is that measurements were restricted to two postoperative time points. During implementation of the study, specific measurement time points were pre-established to reduce dropout rates. Previous research has indicated that patients may withdraw consent from studies due to work interference, time constraints, study demands, and inflexibility [44]. Consequently, it was decided not to include an excessive number of follow-up measurement in the registry. Another limitation of our study is that not all parameters could be included in the multiple regression analysis. As described previously, the postoperative outcome depends on many different clinical and non-clinical factors. However, to answer our hypothesis, we have recorded the most important parameters and integrated them into the regression models. In accordance with the a priori performed sample size calculation, all presented results are valid and not underpowered.

## Conclusion

This is the first study to describe the relationship between HL and postoperative HRQoL in patients with hip OA undergoing THA. The concept of evaluating patient knowledge appears to be a novel aspect of HL. Our results showed that not knowledge but the patients’ satisfaction with the provided information is a short-term predictor of HRQoL. These findings contribute to a new field of research on the interaction between patients’ cognitive abilities to process information and postoperative outcomes. For the clinical practice, the results imply that surgeons should not only provide knowledge about the disease and surgery but should also ask about the patient’s satisfaction with the received information to improve outcomes. From a scientific perspective, there are several opportunities for future research. Prospective randomized intervention studies could evaluate targeted education interventions to improve HL and patient satisfaction to gain insights into the relationship between these factors and HRQoL. Evaluating the impact of tailored interventions on patient-reported outcomes could support the development of evidence-based strategies to improve quality of life after THA. Developing targeted education strategies that focus on improving disease-specific knowledge and preoperative information may help optimize patient satisfaction and HRQoL outcomes in the early postoperative period. In summary, these future approaches have the potential to expand our understanding of the complex interplay between HL, patient satisfaction and HRQoL following THA.

### Supplementary Information

Below is the link to the electronic supplementary material.

Supplementary file1 Supplementary Figure 1. English version of the knowledge quiz on prosthesis and surgery (PDF 84 KB)

Supplementary file2 Supplementary Figure 2. Mean and standard deviation of all outcome variables and their Pearson-intercorrelation (PDF 251 KB)

## Data Availability

The data that support the findings of this study are available from the corresponding authors [AS/TR] upon reasonable request.

## Declarations

### Clinical trial registration

In the context of a retrospective evaluation of a clinic's own registry a trial registration was waived.
