# Supplementary material for: Influence of health literacy on health-related quality of life after total hip arthroplasty
Source: Arch Orthop Trauma Surg. 2023 Oct 26;144(3):1389–400. doi: 10.1007/s00402-023-05098-0 (PMC10896873; doi:10.1007/s00402-023-05098-0)
Supplement: Supplementary file 1 — Supplementary file1 Supplementary Figure 1. English version of the knowledge quiz on prosthesis and surgery (PDF 84 KB) [file 402_2023_5098_MOESM1_ESM.pdf]

KNOWLEDGE QUIZ ON PROSTHESIS AND SURGERY

The following questions are intended to determine your knowledge about the upcoming surgery and your disease. If you are unsure about a question, please select the answer that you think applies best. Please do not omit any question.

1. Please name three of the most important complications of total hip arthroplasty:

(1) …………………………………………………………………………………………

(2) …………………………………………………………………………………………

(3) …………………………………………………………………………………………

The following questions refer to the topic "Hip joint and Arthroplasty". In each line, please mark whether the statement is false or true.

**1.** Which of the following bones form your hip joint?	**true**	**false**
**a.** Acetabulum	O	O
**b.** Femoral neck	O	O
**c.** Ilium	O	O
**d.** Femoral head	O	O
**2.** Which of the following items are risk factor that increase your postoperative risk of infection?	**true**	**false**
**a.** Physiotherapy	O	O
**b.** Diabetes	O	O
**c.** Alcohol consumption	O	O
**d.** Smoking	O	O
**e.** Overweight	O	O
**3.** Which of the following steps are mandatory during surgery to successfully implant the prosthesis?	**true**	**false**
**a.** Milling out the acetabulum	O	O
**b.** Gentle detachment of muscles	O	O
**c.** Gentle transection of the sciatic nerve	O	O
**d.** Saw off the femoral head	O	O

**4.** Which of the following items are specific risks after total hip arthroplasty, i.e., risks attributable to the prosthesis?	**true**	**false**
**a.** Leg length differences	O	O
**b.** Asthma	O	O
**c.** Muscular dysbalance	O	O
**d.** Dislocation of the hip joint	O	O
**e.** There are no specific risks	O	O
**5.** What can positively influence the recovery after arthroplasty in the first weeks?	**true**	**false**
**a.** Adequate amount rest and careful mobilisation during the first days after arthroplasty	O	O
**b.** Cooling of the operated joint	O	O
**c.** A lot of running, exercise and sports	O	O
**d.** Physiotherapy	O	O
**e.** Protein-rich and vitamin-rich	O	O
**6.** Which personal behaviour can optimise the survival of the hip prothesis?	**true**	**false**
**a.** Physical activity adapted to the hip arthroplasty	O	O
**b.** Avoiding carrying of heavy loads	O	O
**c.** Increased use of public transport	O	O
**d.** Avoiding overloading of the hip	O	O

The following questions each have **multiple answer options**, but only one of them is correct. Please **mark only one** answer per question.

1. What is the average postoperative infection risk of arthroplasty?

O less than 1%
O 5% to 10%
O 1% to 4%
O more than 10%

2. On average, how long does a hip replacement last?

O 5 to 10 years
O 15 to 20 years
O 10 to 15 years
O 20 to 25 years

3. To what extent are you usually allowed to put weight on your hip after hip arthroplasty?

O Pain-adapted full load
O Partial load of 20 kg
O Partial load of 10 kg
O Complete relief, i.e. no weight-bearing possible
