# Supplementary figures and images for: Influence of health literacy on health-related quality of life after total hip arthroplasty
Source: Arch Orthop Trauma Surg. 2023 Oct 26;144(3):1389–400. doi: 10.1007/s00402-023-05098-0 (PMC10896873; doi:10.1007/s00402-023-05098-0)

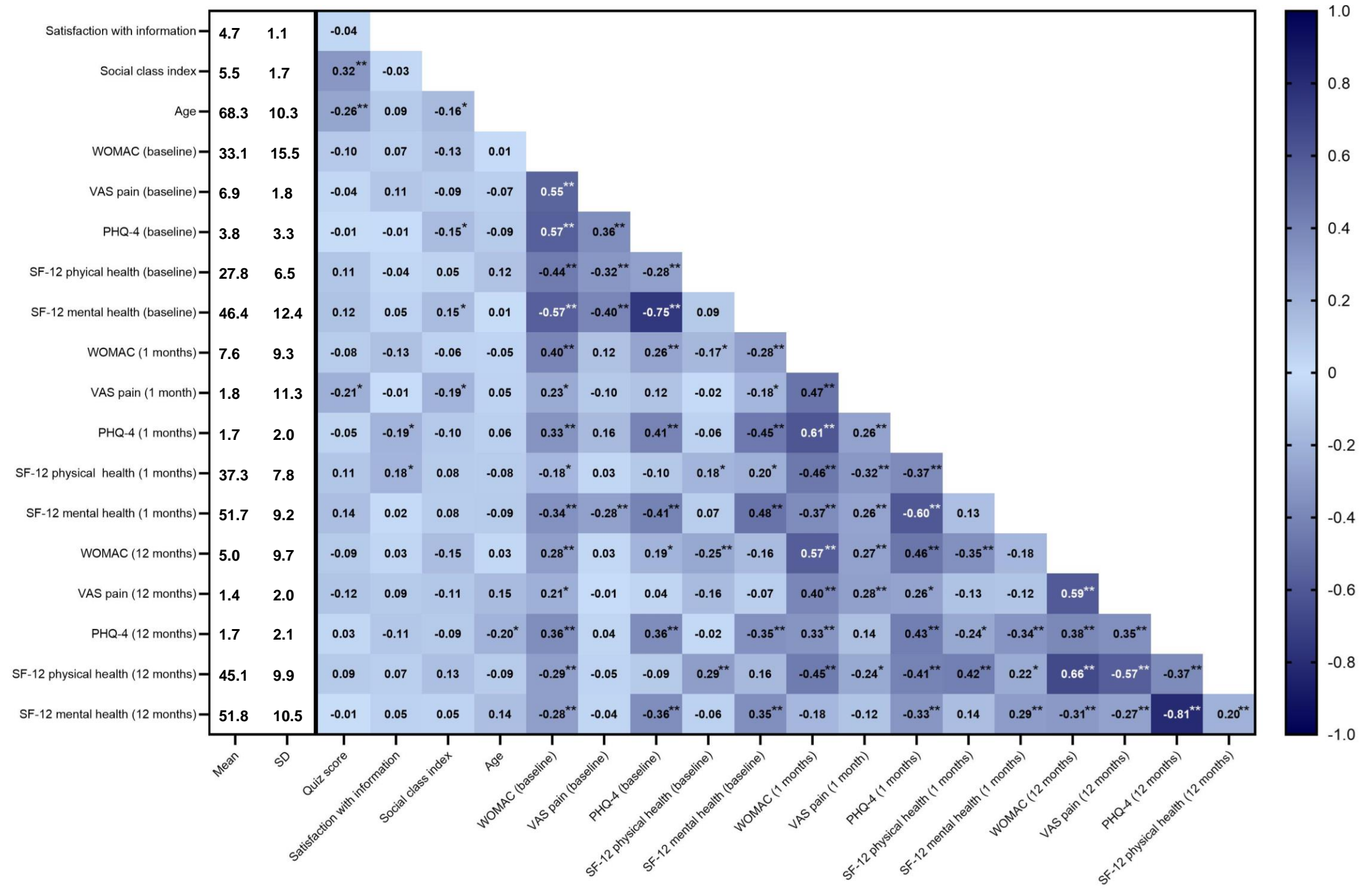

Supplement: Supplementary file 2 — Supplementary file2 Supplementary Figure 2. Mean and standard deviation of all outcome variables and their Pearson-intercorrelation (PDF 251 KB) [file 402_2023_5098_MOESM2_ESM.pdf]
